# Paper-based inkjet bioprinting to detect fluorescence resonance energy transfer for the assessment of anti-inflammatory activity

**DOI:** 10.1038/s41598-017-18995-3

**Published:** 2018-01-12

**Authors:** Annie Agnes Suganya Samson, Jungmi Lee, Joon Myong Song

**Affiliations:** College of Pharmacy, Seoul National University, Seoul, 08826 South Korea

## Abstract

For the first time, a paper-based fluorescence resonance energy transfer (FRET) determination with cyclic AMP (cAMP)-specific phosphodiesterase 4B (PDE4B) inhibitory assay using an inkjet-printing technique is proposed. Non-fabricated parchment paper is found to constitute a unique substrate to measure fluorescent energy transfer, due to its insignificant self-absorption, and enables efficient sample interaction. Here, we report the responsive FRET signals generated on paper, upon sequentially printing reaction components on parchment paper using a conventional inkjet printer equipped with four cartridges. After printing, the energy emitted by Eu chelate was transferred by FRET to ULight molecule on paper, detected at 665 nm. In the absence of free cAMP, a maximum FRET signal was achieved on paper, while a decrease in FRET signals was recorded when free cAMP produced by PDE4B inhibitors compete with Eu-cAMP, binding with ULight-mAb. The IM_50_ value was determined as 2.46 × 10^−13^ mole for roliparm and 1.86 × 10^−13^ mole for roflumilast, to effectively inhibit PDE4B activity. Inkjet printing-based FRET signal determination utilizes components that are less than the femtomole range, which was four-orders less than the standard assay method. The methodology reported here constitutes an innovative approach towards the determination of FRET signals generated on paper.

## Introduction

In the past few years, paper-based inkjet printing biosensor and bioanalytical tools have been extensively used for the rapid detection of biomolecule interactions. Many research activities increasingly concentrate on microfluidic devices fabricated with glass and polymer surfaces, which have attracted great attention because of their potential miniature form and automation. Specifically, studies have validated these paper-based microfluidic biosensors, i.e., surface-modified miniaturized microfluidic devices, as a novel analytical tool for sequential analytical measurements. It includes chemiluminescent methods^1^, surface-Raman spectroscopy^2^, electrochemical^3^, and FRET-based fluorescent detection methods^4–6^. On the other hand, the design and fabrication of these microfluidic devices would be complex, highly expensive, and time- consuming. Hence, a growing need exists for a cost-effective and similar method for the detection of biomolecules. Nitrocellulose (NC) membranes^7,8^, filter paper^9^, parchment paper^9^, chromatographic paper^8^, or glass fiber paper-like^6^ substrate have been utilized as the paper-based material, because they possess high protein/enzyme binding capability that makes it available for bio-molecular immobilization. These porous membranes or paper operates based on capillary action to transport and react with liquid samples. So far, paper-based inkjet printing microfluidic sensors have been employed for sandwich ELISAs^7^, α-amylase detector for disease diagnosis^10^, detection of acetylcholinesterase (AChE) inhibitors^11^, and micro-colorimetric biochemical (glucose/glucose oxidase, DNA/hydrogen peroxidase and biotin/streptavidin) detection method^12^. Similarly, automated paper-based inkjet printing sandwich ELISA was fabricated on a piece of nitrocellulose membrane to analyze human chronic gonadotropin (hCG). However, this method also includes multiple steps, and crucial printing patterns are required to obtain quantitative outcomes^7^. Different paper-based microfluidic devices have reported FRET-based fluorescent assay for the direct detection of protein, nucleic acid, and upconversion phosphors (UCPs) suitable for molecular diagnosis^4,6^. Recently, portable paper-based sensor bis (dithiocarbamato) copper (II) complex functionalized carbon nanodots (CDs) for the detection of mercuric ion (Hg^2+^) were developed by printing CuDTC2-CD solution on cellulose acetate paper using a commercial inkjet printer^5^. Although various inkjet-printing paper-based ELISA platforms with colorimetric detection for drug screening, and molecular diagnosis and enzyme inhibitory analysis have been successfully developed in the recent years, a paper-based inkjet-printing technique is still not widely applied for FRET detection. Paper-based assays are commonly utilized for detecting biologically small molecules and macromolecules because of their effective accessibility and fewer false-positive results^13–16^. A variety of applications based on paper and inkjet printing-based diagnosis have been reported for molecular diagnosis^17–23^, RNA detection and analysis for Ebola virus diagnosis^13^, C-reactive protein (CRP) monitoring^24^, multiplexed point-of-care diagnostic devices to detection of nucleic acids, malaria and dengue^14–16,25,26^.

In the present study, for the first time, fluorescence resonance energy transfer (FRET) determination with cyclic AMP (cAMP)-specific phosphodiesterase 4B (PDE4B) inhibitory assay using an inkjet-printing technique is proposed. FRET signal measures the interaction between two molecules labeled with two different fluorophores (i.e., the donor and the acceptor), by the transfer of energy from the excited donor to the acceptor. Various methods are available in the extent literature to quantify and plot the FRET signal^27^, but the measurement that they offer involves numerous practical difficulties, including calculation error, difficult interpretation, and high sensitivity. We propose here a quantitative method by using a non-fabricated parchment paper surface to measure the FRET system with controlled amounts (nanoliter volume) of donor and acceptor fluorophores using a conventional inkjet printer equipped with four cartridges. The reaction sample solutions, including cAMP, PDE4B, roliparm or roflumilast, Eu- anti cAMP, and ULight cAMP are sequentially printed on parchment paper through a layer-by-layer process. This paper demonstrates successful completion between Eu chelate- labeled cAMP tracer (donor) and ULight- anti-cAMP dye (acceptor) on parchment paper. After printing, Eu chelate- labeled cAMP tracer is excited, and the energy emitted by Eu chelate was transferred by FRET to ULight molecule on paper, detected at 665 nm using a fluorescent microscope. In the absence of free cAMP maximum, the FRET signal was achieved on paper, while a decrease in the FRET signal was recorded when free cAMP produced by PDE4B inhibitors compete with Eu-cAMP, binding with ULight-mAb. Parchment papers are found to be a unique substrate to measure fluorescent energy transfer, due to their insignificant self-absorption, which facilitates efficient interaction of reaction components. This new parchment paper-based enzyme-inhibitor interaction/FRET assay offers several major advantages, including low reagent consumption, cost-effectiveness, no need of a fabrication process, stability, and biodegradability. In contrast to standard FRET measurements, the inkjet printing-based FRET determination precisely evaluates the inhibitory mole 50 (IM_50_) with an effective number of mole of PDE4B inhibitor printed per spot. IM_50_ is the amount of inhibitor used to react with different concentrations of substrate printed on paper. Hence, the methodology reported here constitutes an innovative approach towards the quantitative determination of FRET signals generated on paper.

## Results

### Schematic diagram

**Figure 1:**
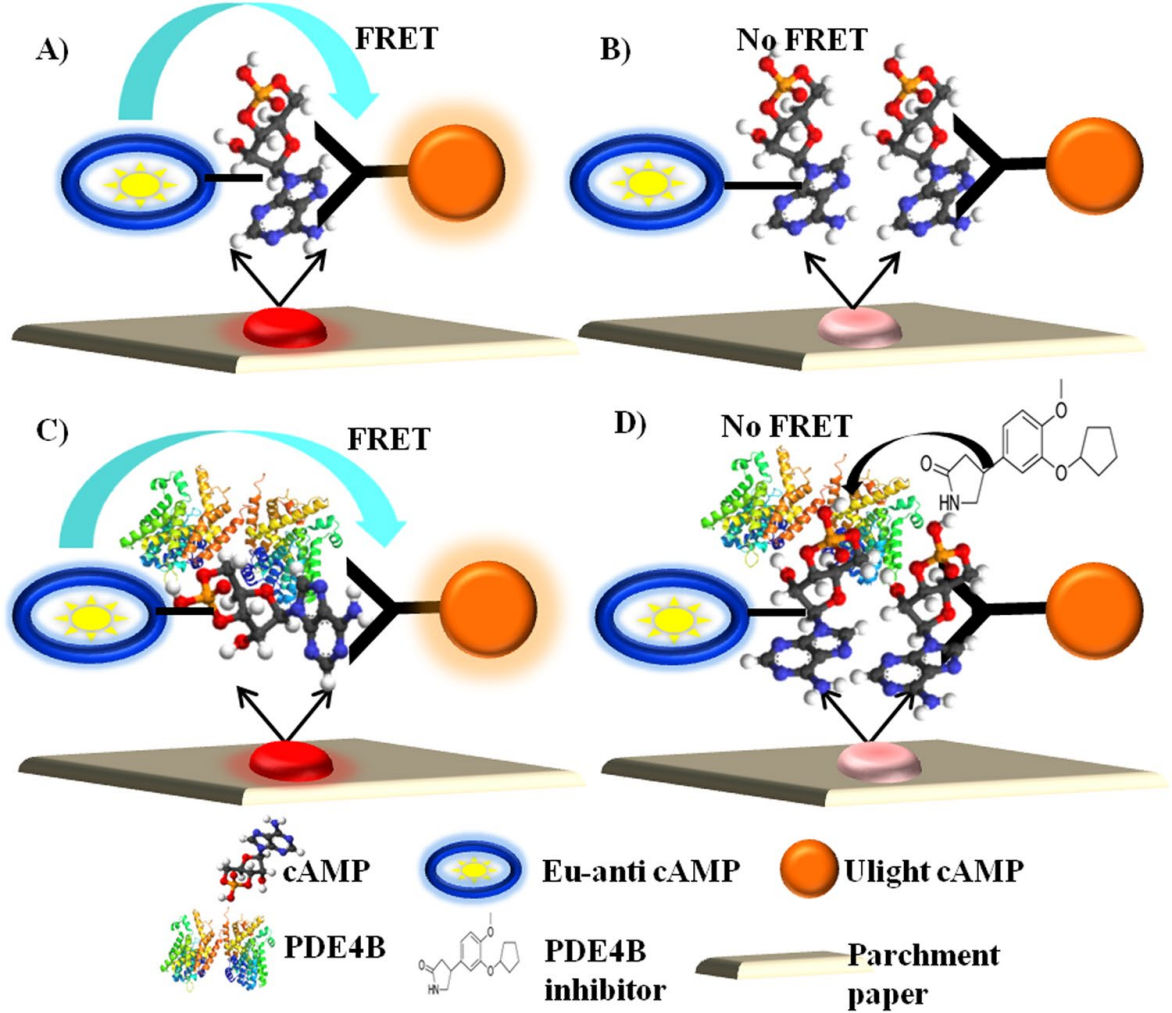
Schematic diagram. The schematic diagram shows that the reaction components were printed on parchment paper, and the fluorescence energy transfer between Eu-cAMP and ULight-anti-cAMP was determined under four different conditions, including: (**A**) absence of free cAMP; (**B**) presence of free cAMP; (**C**) presence of cAMP and PDE4B enzyme; and (**D**) presence of cAMP, PDE4B enzyme, and PDE4B inhibitor.

PDE4B is a member of the phosphodiesterase family of proteins, which plays a critical role in regulating intracellular levels of cyclic AMP (cAMP)^28^. cAMP-specific 3′,5′-cyclic phosphodiesterase 4B is a therapeutic target for the treatment of several inflammatory disorders^29^. Here, we present the development of a simple, cost-effective, less time-consuming, and highly specific PDE4B assay on a non-fabricated parchment paper surface using inkjet-printing technology. Mainly, this assay is based on the competition between the europium (Eu) chelate-labeled cAMP tracer and sample cAMP for binding sites on cAMP-specific monoclonal antibodies (mAb) labeled with the ULight dye on parchment paper. PDE4B enzyme and their respective inhibitors (roliparm and roflumilast)^30,31^ were utilized to study FRET-based enzyme-inhibitor interactions on paper. In connection with this, Fig. 1 represents the scheme of the present study. When ULight-monoclonal antibody is bound to the Eu-labeled cAMP tracer, a light pulse at 320/340 nm excites the Eu chelate molecule of the cAMP tracer. The energy emitted by the excited Eu chelate is transferred by FRET to ULight molecules on the antibodies, which in turn emit fluorescence at 665 nm. Residual energy from the Eu chelate will produce fluorescence at 615 nm. After printing the reaction components on parchment paper, fluorescence energy transfer between Eu-cAMP and ULight-anti-cAMP provides a specific binding signal in the absence of free cAMP (Fig. 1A), while energy transfer does not occur in the presence of free cAMP (Fig. 1B). FRET between Eu-cAMP and ULight-anti-cAMP provides a particular signal, that is utilized here as an optical method to quantify cAMP-specific PDE4B and their respective inhibitor interactions on paper. In the presence of PDE4B, the cAMP is degraded into AMP, which is not recognized by the ULight-mAb. This leads to an increase in FRET signal, which is proportional to the concentration of degraded cAMP (Fig. 1C). Similarly, in the presence of PDE4B inhibitor, cAMP remains intact and competes with the Eu-cAMP tracer for binding to the Ulight-mAb. This results in a decrease in FRET signal, which is proportional to increased inhibitor concentration (Fig. 1D).

### Optimization of cAMP activity using an inkjet-printing method

**Figure 2:**
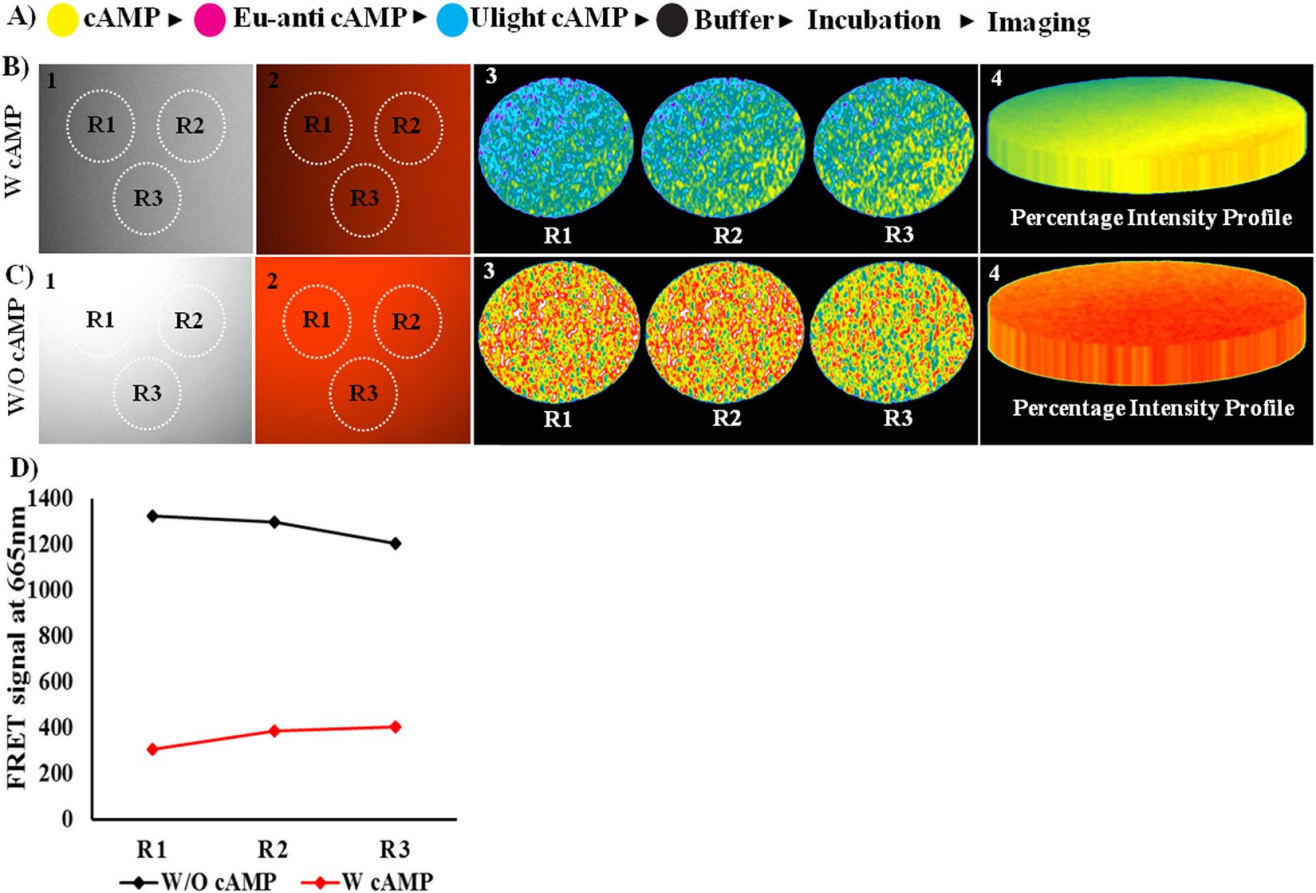
Optimization of cAMP activity using the inkjet-printing method. (**A**) Represents printing order. (**B**) FRET signals were acquired with cAMP, and their respective images were illustrated as: 1. monochrome image; 2. fluorescent dye color image; 3. intensity profile of three different regions; and 4. percentage intensity profile of three regions. (**C**) FRET signals were acquired without cAMP, and their respective images were shown as: 1. monochrome image; 2. fluorescent dye color image; 3. intensity profile of three different regions; and 4. percentage intensity profile of three regions. (**D**) Fluorescence signal generated on a single spot was measured. The graph illustrates the individual fluorescence signal produced with and without cAMP by three different regions/spot areas.

Firstly, optimal cAMP activity was measured to evaluate the cAMP-specific PDE4B enzyme inhibitory assay. The spot size 0.3 cm with an area of 0.071 cm^2^/spot was used for printing. The cAMP (Y cartridge), Eu-cAMP tracer (M cartridge), ULight anti-cAMP mAb (C-cartridge), and Buffer (K cartridge) were sequentially printed on parchment paper based on a layer-by-layer process using a conventional inkjet printer (Fig. 2A). Two different sets of experiments (W cAMP and W/O cAMP) were performed to verify cAMP activity in the presence of Eu-cAMP tracer and ULight anti-cAMP mAb. The reaction components were printed on parchment paper in reference to Fig. 2A. Hence, two different spot areas were printed, W and W/O cAMP. Immediately, Eu-cAMP tracer and ULight anti-cAMP mAb were printed on the cAMP pre-printed spot surface and incubated for 30 min (Fig. 2B,C). Competitive fluorescent energy transfer occurred in the presence and absence of cAMP measured at the 665-emission range using a fluorescent microscope, and the FRET images were analyzed with MetaMorph software. Figure 2B(1 and 2) and 2C(1 and 2) represent the monochrome and fluorescent dye color image of the FRET signal acquired with W and W/O cAMP, respectively. Three different regions (R1, R2, and R3) were selected to confirm the uniform FRET signal distributed on parchment paper, and their representative individual intensity profiles and percentage intensity profiles are shown in Fig. 2B(3 and 4) and 2C(3 and 4), respectively. Individual and average FRET signals corresponding to R1, R2, and R3 that acquired W and W/O cAMP are graphically represented as Fig. 2D.

Although significant FRET signals were acquired with W and W/O cAMP, it is important to assess the amount of cAMP involved in the reaction. In our previous work, we developed a novel method using an inkjet printer to evaluate the number of mole printed on the surface based on volume printed per spot and their respective solution density^32^. This approach enhanced the application of the inkjet-printing technique to study enzyme inhibitory assay. The described method^32^ was applied in the present study to evaluate the number of mole of cAMP printed on paper. Initially, the reaction sample solution densities were evaluated using a pycnometer and expressed in g/mL (Supplementary Table S1). The printed volume was evaluated using solution densities, and cartridge weight loss before and after printing. Thus, the printed volume was calculated using the formula: printed volume = (cartridge weight loss/solution density) * area. Supplementary Table S2 shows that 27.8 nL of cAMP was printed/spot area to react with 30 nL of Eu-cAMP tracer and 30 nL of ULight anti-cAMP mAb, which were printed on the same spot area. The effective mole printed on the surface was estimated using estimated printed volume and initial solution stock concentration using a standard equation: number of mole = molarity * volume. It was determined that 2.79 × 10^−16^ mole of cAMP was printed per spot area.

### Optimization of PDE4B enzyme activity using an inkjet-printing method

**Figure 3:**
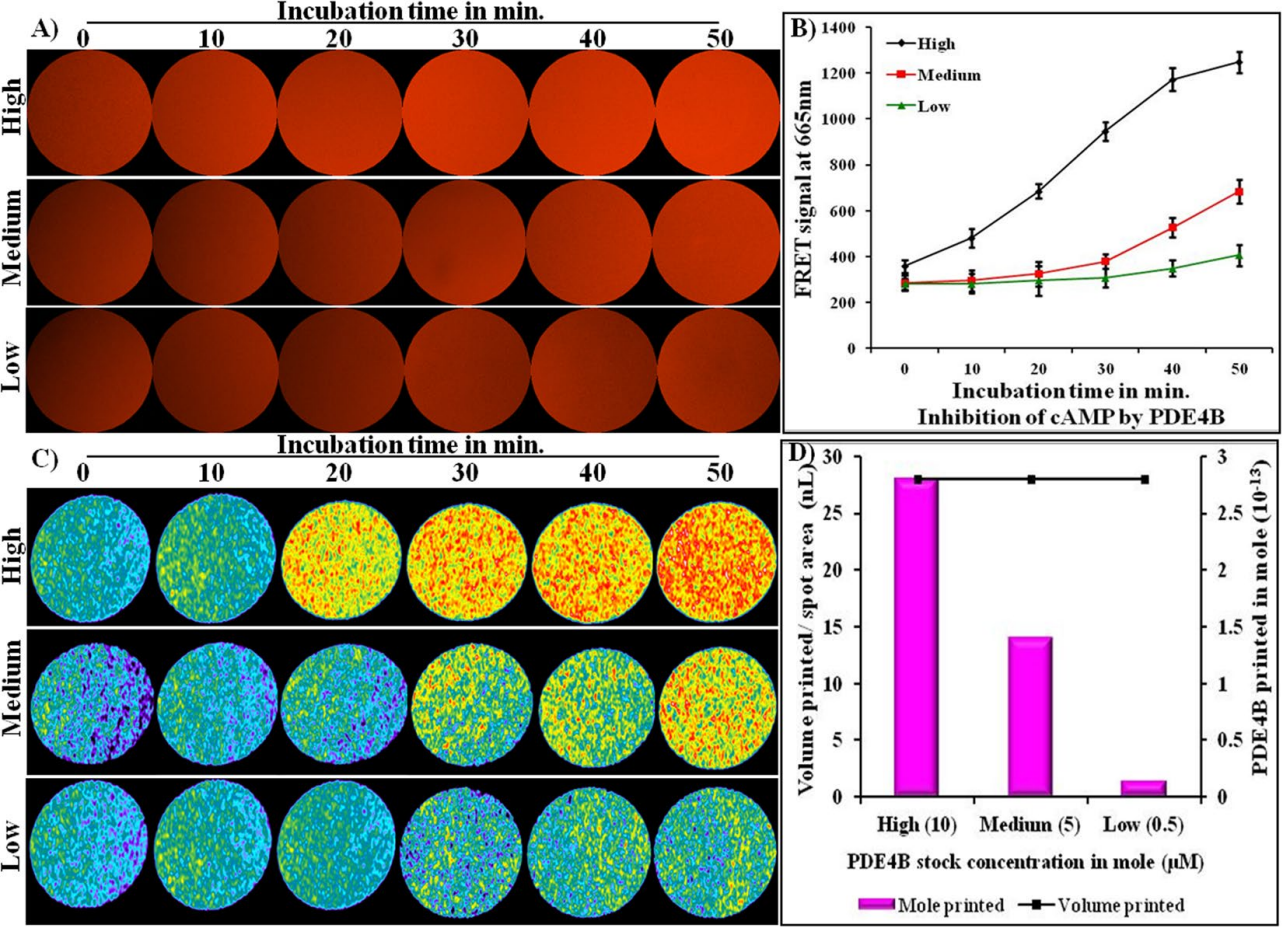
Optimization of PDE4B enzyme activity using the inkjet-printing method. (**A**) Fluorescence signal generated on parchment paper; (**B**) graph represents the average fluorescence intensity of the individual images (3A); (**C**) illustrates the intensity profile of the individual images (3A). Figure data A, B, and C were acquired as a function of dose and time point; and (**D**) the X-axis represents the number of mole of PDE4B printed per spot, the primary Y-axis represents the FRET signals produced in the presence of cAMP and PDE4B, and the secondary Y-axis represents the ejection volume of PDE4B.

Inkjet bioprinting was performed and PDE4B enzyme reaction curve was obtained at three different concentrations (stock concentrations: high −10 µM, medium −5 µM, and low −0.5 µM) using 2.79 × 10^−16^ mole of cAMP as a substrate on parchment paper. cAMP, PDE4B, Eu-cAMP tracer, and ULight anti-cAMP mAb were sequentially printed on parchment paper based on a layer-by-layer process using a conventional inkjet printer. Figure 3 illustrates the optimization condition under which the FRET signal correlates linearly with time. As shown in Fig. 3A,B, the signal increased significantly as a function of time up to 50 min with a higher concentration of PDE4B; whereas, the signal reached a plateau after only 30 min when medium and low concentration of enzyme were used. Figure 3A represents the FRET images acquired using a fluorescent microscope, and Fig. 3B corresponds to their respective fluorescent intensity determined using Metamorph software. Figure 3C presents the intensity profile of fluorescent signals produced on parchment paper, which confirms the linear correlation between the fluorescent signal and time point. The intensity profile image clearly supports that the FRET signal increased linearly as a function of time up to 50 min with a higher concentration of PDE4B. Similarly, non-linear signals were generated on paper in the presence of medium and low concentration of PDE4B (Fig. 3C). As previously mentioned, the volume printed and number of mole of PDE4B printed per spot area were evaluated. The number of mole of PDE4B printed per spot area corresponds to 2.81 × 10^−13^ mole, 1.40 10^−13^ mole and 1.40 10^−14^ mole, which was ejected from the cartridges filled with high –10 µM, medium −5 µM, and low −0.5 µM concentration of PDE4B, respectively (supplementary Table S2). In Fig. 3D, the graphical plot presents the FRET signal generated in response to the enzymatic reaction on paper. The optimized condition here verifies that 2.81 × 10^−13^ mole of PDE4B printed/spot degrading 2.79 × 10^−16^ mole of cAMP leads to an increase in FRET signal, while a significantly lesser FRET signal was generated in the presence of 1.40 10^−13^ mole and 1.40 10^−14^ mole of PDE4B. In order to develop a paper-based sensitive assay and ensure cAMP activity using inkjet-printing technique, 2.81 × 10^−13^ mole of PDE4B and reaction time of approximately 50 min were selected for subsequent experiments.

### Paper-based inkjet bioprinting to determine PDE4B inhibition by roliparm/roflumilast

**Figure 4:**
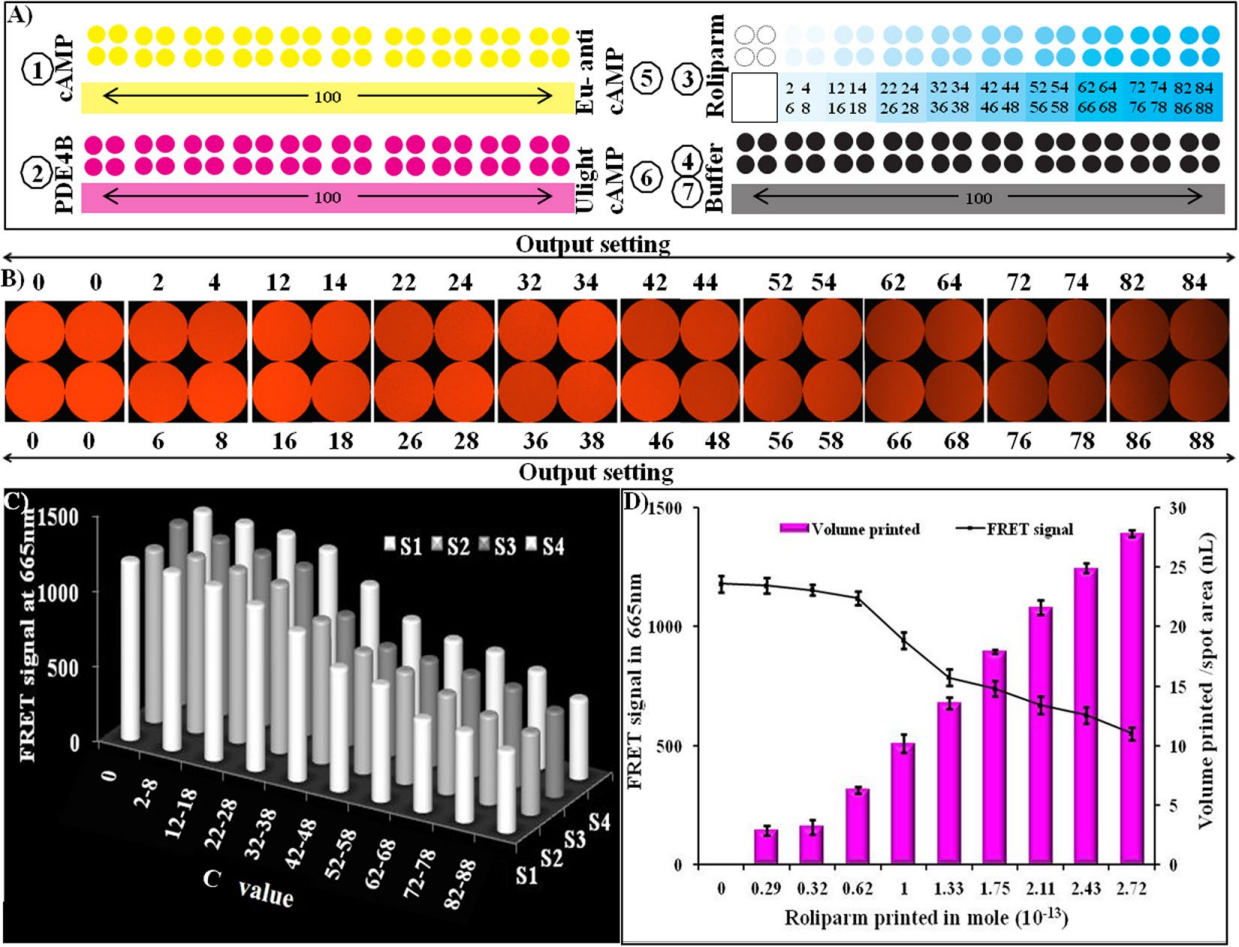
Inkjet-printing based determination of PDE4B inhibition by roliparm. (**A**) Represents printing order; (**B**) fluorescence signal generated on parchment paper as a function of C value; (**C**) graph represents the average fluorescence intensity of the individual images (4A) as a function of C value; and (**D**) the X-axis represents the number of mole of roliparm printed per spot, the primary Y-axis represents the FRET signals produced in the presence of cAMP, PDE4B and roliparm, and the secondary Y-axis represents the ejection volume of roliparm. Error bars represent the standard deviation of three independent measurements.

**Figure 5:**
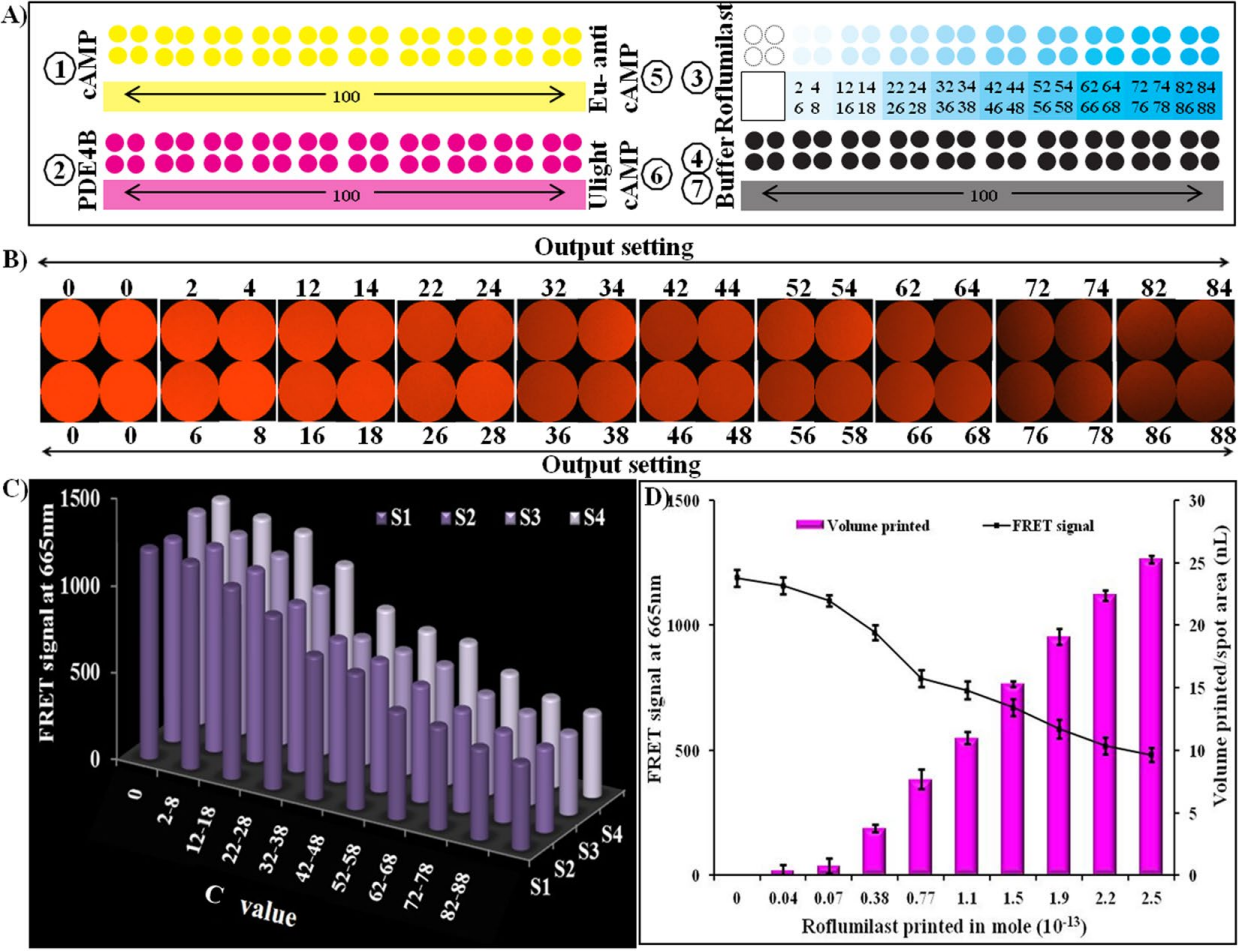
Inkjet-printing based determination of PDE4B inhibition by roflumilast. (**A**) Represents printing order; (**B**) fluorescence signal generated on parchment paper as a function of C value; (**C**) graph represents the average fluorescence intensity of the individual images (4A) as a function of C value; and (**D**) the X-axis represents the number of mole of roliparm printed per spot, the primary Y-axis represents the FRET signals produced in the presence of cAMP, PDE4B and roflumilast, and the secondary Y-axis represents the ejection volume of roflumilast. Error bars represent the standard deviation of three independent measurements.

On the basis of the sensitivity and specificity studies, the practical application of the inkjet printing-based system for determining PDE4B inhibitory activity was investigated. For this purpose, the printing-output setting was fixed as 100, to print cAMP, PDE4B, Eu-cAMP tracer, ULight anti-cAMP mAb, and buffer. In addition, the printing-output setting (C value) was altered by a two-order difference per spot (from 0 to 88) to vary the amount of roliparm or roflumilat to be printed per spot area. Figure 4A(1)–A(4) and Fig. 5A(1)–A(4) show the response order of printing cAMP, PDE4B, and roliparm/roflumilast. Finally, the buffer was printed to avoid drying. Reaction samples printed-paper was incubated for 30 min inside of an airtight container. Immediately, Eu-cAMP tracer and ULight anti-cAMP mAb (Fig. 4A(6) and (7) and Fig. 5A(6) and (7)) were printed from different cartridges on the same spot area, and incubated for 50 min. FRET signals were detected at 665 nm using a fluorescent microscope.

The fluorescent signals were generated as an effect of enzyme inhibitors. Roliparm (Fig. 4B)/roflumilast (Fig. 5B) spotted per area shows that the decrease in FRET signals is directly related to the increase in PDE4B inhibitors spotted on same spot surface area. Individual acquired images clearly verify the variance for roliparm (Fig. 4B)/roflumilast (Fig. 5B) printed per spot with respect to the printing-output settings (C value). In order to classify FRET signals and their corresponding C values; fluorescent images were categorized as S1 (2, 12, 22, 32, 42, 52, 62, 72, 82), S2 (4, 14, 24, 34, 44, 54, 64, 72, 84), S3 (6, 16, 26, 36, 46, 56, 66, 76, 86), and S4 (8, 18, 28, 38, 48, 58, 68, 78, 88). Figure 4C and Fig. 5C represent the fluorescent signal generated on paper, where the X-axis represents the C value and the Y-axis represents the FRET signal. The graph clearly shows a linear reduction in the FRET signal, confirming the PDE4B enzyme inhibitory activity on paper. As previously mentioned, to perform the PDE4B inhibitory assay, 2.79 × 10^−16^ mole of cAMP and 2.81 × 10^−13^ mole of PDE4B were printed on parchment paper. It was found that 0.29 × 10^−13^ mole to 2.72 × 10^−13^ mole of roliparm and 0.04 × 10^−13^ mole to 2.5 × 10^−13^ mole of roflumilast were printed on the surface. Reproducibility of FRET signal on the parchment paper was confirmed by measuring the FRET signals of 3 different sets using three different inkjet-printed parchment papers with same parameters. A graph was plotted to corroborate the volume of roliparm/roflumilast, mole printed per spot area, and their corresponding FRET signals generated on paper. As shown in Fig. 4D and Fig. 5D, the X-axis represents mole of roliparm/roflumilast printed per spot area, and the primary Y-axis represents the volume of roliparm/roflumilast printed per spot area and the secondary Y-axis represents the resultant FRET signals produced on parchment paper. Error bars in the plot represent the standard deviation of three independent measurements. The inhibitory mole 50 (IM_50_) of roliparm and roflumilast, which is required to reduce the PDE4B activity by half, was determined as 2.46 × 10^−13^ mole and 1.86 × 10^−13^ mole, respectively (supplementary Table S3).

## Discussion

cAMP-specific 3′,5′-cyclic phosphodiesterase 4B (PDE4B) was found to constitute an excellent therapeutic target. Inhibition of PDE4B suppresses inflammation^33^. Roliparm and roflumilast were reported as potential PDE4B inhibitors for the treatment of inflammation^34–36^. Although different methods, including, ELISA, gene cloning and radioisotope/alumina acid methods, are available to screen PDE4B inhibitors, these methods are still highly time-consuming. For example, cloning usually takes more than two weeks to establish PDE4B gene-expressing vectors. Consequently, even more time is required for compound screening. Likewise, to perform drug screening using radioisotope/alumina acid methods, handling a liquid system and preparation of alumina plates is inconvenient^37–40^. In comparison, the inkjet printing-based screening approach offers a user-friendly method and is much less time-consuming. The fundamental strategy in this work utilizes non-fabricated parchment paper as a substrate to sense PDE4B inhibitor activity, and in turn, this sensing event produces FRET signals. We present an alternative system, which will substantially enhance the ability to screen future FRET signal-based assay for novel analyses. Our study involves paper-based inkjet bioprinting for FRET signal determination via evaluating the competition between Eu-labelled cAMP tracer and cAMP for binding sites on cAMP- specific mAb labeled with ULight. After printing the reaction components on parchment paper, antibodies bound to the Eu-labelled cAMP. When Eu-chelate molecules were excited at 320/340 nm, the energy is transferred by FRET to ULight molecules, which in turn emitted fluorescence at 665 nm. cAMP-specific PDE4B inhibition results in the production of FRET-based antibody interaction, which displayed a uniquely characteristic low-background signal on parchment paper. Therefore, FRET signals determined at a lower optical background enables us to analyze threshold values sensitively. The experimental results clearly confirm the compatibility and applicability of this inkjet-printing system for quantitative FRET determination. Previous studies have reported an inkjet printing-based multi-enzyme printing system (ADH–DP), to print enzyme and substrate on a piece of parchment paper for certain applications, such as biosensing^9^. Our previous study proposed a novel concept IM_50_ for the quantitative determination of inhibitor efficacy using a conventional inkjet printer containing four cartridges^32^. In addition, earlier reports have verified that gradient color codes preset on a computer can be utilized to quantitatively print enzymes, proteins, and inhibitors on paper. This basic background allowed us to predict the number of mole of component used to react with enzymes/inhibitors. It was also found that a controlled spot size and pattern design played critical roles in creating a paper-based enzymatic assay^9,32^.

Earlier, conventional methods-based cAMP-dependent PDE4B assay was found to be highly sensitive for drug screening, and the optimal working range of cAMP required to inhibit the activity of PDE4B enzyme was determined to be 0.5 µM/1 µM. Under this experimental condition, the IC_50_ value to inhibit PDE4B activity by roliparm and roflumilast was determined as 132 nM and 0.8 nM, respectively^41,42^. In the present study, a commercially available inkjet printer equipped with four cartridges was used to achieve an efficient outcome of PDE4B inhibitor activity against PDE4B enzyme in a dose-dependent manner. Gradient color codes were utilized to print gradient concentration of inhibitors per spot area. To obtain the highest sensitivity and reproducibility, the number of moles of cAMP and PDE4B were optimized using the inkjet-printing method. Our results confirm that Eu-cAMP recognizes ULight anti-cAMP in the absence of cAMP, which was confirmed by the increased FRET signal. However, cAMP remains intact and competes with the Eu-cAMP tracer for binding to the Ulight-mAb in the presence of cAMP, and thus decreases in FRET signals were recorded. Since ejection volume to be printed per spot can be controlled by adjusting the printing-output settings, the number of mole printed per spot can be evaluated. It was determined that 2.79 × 10^−16^ mole of cAMP successfully competes with 30 nL of Eu-cAMP tracer and ULight anti-cAMP mAb printed per spot. This optimal condition for producing the highest fluorescent intensity was selected. Similarly, three different concentrations of PDE4B were utilized to screen PDE4B activity using the inkjet printer. Here, 2.81 × 10^−13^ mole of PDE4B was utilized to interact with 2.79 × 10^−16^ mole of cAMP, where Eu-cAMP was accessible to react with ULight anti-cAMP. The maximum fluorescent signal on parchment paper was achieved in 50 min reaction time. The fluorescent signal was directly proportional to the FRET signal detected in the absence of cAMP. Our results demonstrate that FRET signals were detected on parchment paper at a lower femtomole of cAMP and picomole of PDE4B, which is proven to be more sensitive and reliable than the conventional method.

In our previous work, we observed a gradient increase in the volume used to print per spot according to the gradient increase of the printing setting^32^. Here, the C cartridge was filled with PDE4B inhibitors and the C value was set as the gradient range, and thus the gradient number of moles of roliparm/roflumilast was printed on parchment paper. In contrast, a decrease in fluorescent signal was recorded as the number of mole of inhibitor increased per spot. Paper-based inkjet printing-PDE4B inhibitory assay verifies that 0.29 × 10^−13^ to 2.72 × 10^−13^ mole of roliparm/ 0.04 × 10^−13^ to 2.5 × 10^−13^ mole of roflumilast (PDE4B inhibitor) printed per spot effectively interacts with 2.81 × 10^−13^ mole of PDE4B enzyme and 2.79 × 10^−16^ mole of cAMP, which permits a linear reduction in fluorescent signal. Consequently, from the observed results, it is evident that paper-based inkjet printing-PDE4B inhibitory analysis ensures specific and rapid FRET determination on paper. Compared to conventional well plate method, inkjet printing-based FRET signal determination utilizes a lesser reaction volume up to the nL range. The number of mole of PDE4B inhibitor reacting with PDE4B enzyme significantly verified this, which was four-orders less than the standard assay method. In the presence of cAMP, PDE4B, roliparm/ roflumilast, Eu-cAMP tracer and ULight-anti-cAMP; PDE4B enzyme activity were inhibited by roliparm/roflumilast, while Eu-cAMP recognizes free cAMP. Hence, Eu-cAMP is less available to bind with ULight-anti-cAMP. Conversely, the level of energy transferred to ULight was decreased. Therefore, a significant reduction in fluorescent signal was recorded as a function of the increase in the number of mole of PDE4B inhibitor reacted on paper. The methodology reported here facilitates an innovative approach towards the determination of FRET signals generated on paper. Since this system is relatively inexpensive, readily available and involves simple operating procedures, non-professionals can effectively perform the analysis.

## Conclusion

In summary, we have demonstrated a conventional inkjet-printing system for simultaneous detection of FRET signals and mole of sample consumed in the assay. It utilizes four cartridges as a generalized platform to study enzyme inhibitory activity, whereby FRET signals are generated on parchment paper. The four-cartridge printing system permits determination of mole of enzyme and inhibitor printed per spot as low as the femtomole range. Only a single sample concentration application was required for conducting the entire experiment. Given this, this paper-based enzyme inhibitory assay platform offers the major advantages of low-cost, less sample consumption, consecutive results, and fluorescent signal analysis. We expect that the sensitivity of the experiment can be further improved, and that this approach offers great potential as a novel tool for applicability of FRET determination.

## Methods

### Materials and instruments

Hank’s Balanced Salt Solution (HBSS) (1×) (no pheno red) (Invitrogen), HEPES Buffer Solution (1 M) pH 7.2 to 7.5 (Invitrogen), IBMX (Sigma), cAMP standard, Eu-cAMP tracer, ULight^TM^-anti-cAMP, cAMP detection buffer, and BSA stabilizer were purchased from PerkinElmer. A commercially available HP Officejet Pro 8100 printer was used to print enzyme/inhibitor solutions. This printer was chosen because a high quality of output resolution was certified by the manufacturer, and these cartridges can be easily refilled. Readily available non-fabricated parchment paper was utilized as a substrate in the inkjet printer for the first time. The parchment paper was purchased from Lebus Corporation. The paper is made with 100% pure cellulose fiber. The basic weight and the size is 24 Lb., and 8 ½”x11”, respectively.

### Preparation of solutions and processing of printing cartridges

All of the reagents used in the experiment were freshly prepared, and the experimental procedures were carried out at room temperature under a dark condition. A stimulation buffer consisting of 1 × HBSS, 5 mM HEPES, 0.5 mM IBMX, and 0.1% BSA (pH 7.4) was freshly prepared. cAMP (10 nM) and PDE4B (0.5 µM, 5 µM, and 10 µM) were prepared using the stimulation buffer, and Eu-cAMP tracer (1/150 dilution) and ULight-anti-cAMP (1/300 dilution) were prepared using the cAMP detection buffer. Roliparm (10 µM) and roflumilast (10 µM) were prepared using DMSO. Commercial inks were completely removed, washed with deionized water and 100% ethanol, and then completely air-dried in a hot air oven. Finally, printing solutions were refilled into the respective cartridges.

### Process of Ink deposition

A commercially available HP Officejet Pro 8100 printer was set to operate at normal speed. Adobe Photoshop software was utilized to attain the RGB color code, and the Microsoft Power Point tool was used to attain the desired printing patterns. Solutions of cAMP, PDE4B, Eu-cAMP tracer, ULight anti-cAMP mAb were prepared with stimulation buffer containing 180 μL of PEG-400 and 100 μL of *tert*-butanol per 1 mL. The printing-output setting was fixed as 100, to print cAMP, PDE4B, Eu-cAMP tracer, ULight anti-cAMP mAb, and buffer. The printing-output setting to define ejection volume was altered by two-orders of difference per spot (from 0 to 88) to vary the amount of roliparm or roflumilast. The time interval between printing each element (cAMP, PDE4B, Eu-cAMP tracer, ULight anti-cAMP mAb, and buffer) was optimized as 10 min. Under optimized condition, printing different components layer-by-layer at the same spot created a homogeneous layer that effectively provided the mixing of assay components.

### Assay conditions

To evaluate cAMP-specific PDE4B inhibitor activity, C, M, Y, and K cartridges were refilled with reaction sample solutions. To optimize cAMP activity using a conventional inkjet printer, cAMP (Y cartridge), Eu-cAMP tracer (M cartridge), and ULight anti-cAMP mAb (C cartridge) were sequentially printed on parchment paper, and incubated for 50 min at room temperature (25 °C and a relative air humidity-RH, of ∼50%) inside of an airtight container. To evaluate the optimal PDE4B enzyme concentration using a conventional inkjet printer, cAMP (Y cartridge) and PDE4B (M cartridge) were consequently printed on parchment paper, and incubated for 30 min at room temperature (25 °C and a relative air humidity-RH, of ∼50%) inside of an airtight container. Then, Eu-cAMP tracer (Y cartridge) and ULight anti-cAMP mAb (M cartridge) were sequentially printed on the same spot and then incubated at room temperature. PDE4B-cAMP interaction and their respective FRET signals were detected based on concentration and in a time-dependent manner. Similarly, inhibition of PDE4B enzyme was investigated using the inkjet-printing system. To verify this, cAMP (Y cartridge), and PDE4B (M cartridge), and roliparm/roflumilast (C cartridge) were successively printed on parchment paper, and incubated for 30 min at room temperature inside of an airtight container. Then, Eu-cAMP tracer (Y cartridge) and ULight anti-cAMP mAb (M cartridge) were serially printed on the same spot and then incubated for 50 min at room temperature. Our previous report discussed the impact of measuring solution density and volume printed per spot. Hence, the number of mole printed was evaluated using solution density, and volume printed on the surface, as described by Lee *et al*.^18^. Briefly, solution density was measured by pycnometer, and is expressed as mass per unit volume. The density was calculated using the following formula: ds = (Ws − Wb)/(Ww − Wb), where Ws is solution weight, Wb is bottle weight, and Ww is water weight (supplementary Table S1). Therefore, printed volume was evaluated using cartridge weight loss before and after printing, and solution densities (volume printed = (cartridge weight loss/solution density) *area). The effective number of mole of substance printed on the surface was estimated using printed volume and initial solution stock concentration by means of a standard equation: number of mole = molarity * volume.

### FRET signal measurement

FRET signals generated on parchment paper were analyzed using a fluorescent microscope (OLYMPUS, IX73), and the fluorescent images were captured using a charge-coupled device (CCD) as a function of wavelength. At 320/340 nm, the Eu-cAMP tracer was excited on parchment paper, which was mounted on the sample stage. The laser beam was purified by an interference filter, and it was reflected by a dichromatic mirror and focused on the paper with a 20 × objective lens. The fluorescence emission was transferred to Ulight-anti-cAMP at 665 nm, and was collected by the same microscope objective lens, which passed through a dichromatic mirror and was directed. A charge -coupled device (CCD) camera was used to detect the transmitted fluorescent beam. Light scattering was removed by a filter, which was placed in front of the CCD camera. The background (without sample) and fluorescent (with sample) images were captured. Image analysis was performed using commercially available software, MetaMorph, Version 7.1.3.0 (Molecular Devices). The average threshold values were acquired, and their percentage fluorescence intensity profiles were evaluated using the above-mentioned software.

### Data analysis

Statistical analysis were performed by using OriginPro 8 software.

## Electronic supplementary material

Supplementary Tables

## References

[1] Ge L (2012). Three-dimensional paper-based electrochemiluminescence immunodevice for multiplexed measurement of biomarkers and point-of-care testing. Biomaterials.

[2] Chen YY (2013). A paper-based surface-enhanced resonance Raman spectroscopic (SERRS) immunoassay using magnetic separation and enzyme-catalyzed reaction. Analyst.

[3] Wu YF, Xue P, Kang YJ, Hui KM (2013). Paper-Based Microfluidic Electrochemical Immunodevice Integrated with Nanobioprobes onto Graphene Film for Ultrasensitive Multiplexed Detection of Cancer Biomarkers. Analytical Chemistry.

[4] He MY, Liu ZH (2013). Paper-Based Microfluidic Device with Upconversion Fluorescence Assay. Analytical Chemistry.

[5] Yuan C, Liu BH, Liu F, Han MY, Zhang ZP (2014). Fluorescence “Turn On” Detection of Mercuric Ion Based on Bis(dithiocarbamato)copper(II) Complex Functionalized Carbon Nanodots. Analytical Chemistry.

[6] Li H, Fang XE, Cao HM, Kong JL (2016). Paper-based fluorescence resonance energy transfer assay for directly detecting nucleic acids and proteins. Biosensors & Bioelectronics.

[7] Apilux A, Ukita Y, Chikae M, Chailapakul O, Takamura Y (2013). Development of automated paper-based devices for sequential multistep sandwich enzyme-linked immunosorbent assays using inkjet printing. Lab on a Chip.

[8] Costa, M. N. *et al*. A low cost, safe, disposable, rapid and self-sustainable paper-based platform for diagnostic testing: lab-on-paper. *Nanotechnology* **25**, 10.1088/0957-4484/25/9/094006 (2014). 10.1088/0957-4484/25/9/094006 24521980

[9] Zhang YF, Lyu FJ, Ge J, Liu Z (2014). Ink-jet printing an optimal multi-enzyme system. Chemical Communications.

[10] Dutta S, Mandal N, Bandyopadhyay D (2016). Paper-based alpha-amylase detector for point-of-care diagnostics. Biosensors & Bioelectronics.

[11] Wu Y, Sun YF, Xiao FB, Wu ZY, Yu RQ (2017). Sensitive inkjet printing paper-based colormetric strips for acetylcholinesterase inhibitors with indoxyl acetate substrate. Talanta.

[12] Davaji B, Lee CH (2014). A paper-based calorimetric microfluidics platform for bio-chemical sensing. Biosensors & Bioelectronics.

[13] Magro L (2017). Paper-based RNA detection and multiplexed analysis for Ebola virus diagnostics. Sci Rep.

[14] Piety NZ (2016). Validation of a Low-Cost Paper-Based Screening Test for Sickle Cell Anemia. PLoS One.

[15] Deraney RN, Mace CR, Rolland JP, Schonhorn JE (2016). Multiplexed, Patterned-Paper Immunoassay for Detection of Malaria and Dengue Fever. Analytical Chemistry.

[16] Weaver AA, Lieberman M (2015). Paper Test Cards for Presumptive Testing of Very Low Quality Antimalarial Medications. Am J Trop Med Hyg.

[17] Dincer C, Bruch R, Kling A, Dittrich PS, Urban GA (2017). Multiplexed Point-of-Care Testing - xPOCT. Trends Biotechnol.

[18] Yetisen AK, Akram MS, Lowe CR (2013). Paper-based microfluidic point-of-care diagnostic devices. Lab Chip.

[19] Connelly JT, Rolland JP, Whitesides GM (2015). “Paper Machine” for Molecular Diagnostics. Anal Chem.

[20] Tiwari S, Vinchurkar M, Rao VR, Garnier G (2017). Zinc oxide nanorods functionalized paper for protein preconcentration in biodiagnostics. Sci Rep.

[21] Hosseini, S., Vazquez-Villegas, P. & Martinez-Chapa, S. O. Paper and Fiber-Based Bio-Diagnostic Platforms: Current Challenges and Future Needs. *Appl Sci-Base*l **7**, doi:ARTN 86310.3390/app7080863 (2017).

[22] Nie J (2012). Low-cost fabrication of paper-based microfluidic devices by one-step plotting. Anal Chem.

[23] Hossain SMZ (2009). Development of a Bioactive Paper Sensor for Detection of Neurotoxins Using Piezoelectric Inkjet Printing of Sol-Gel-Derived Bioinks. Analytical Chemistry.

[24] Lin SC (2016). Paper-based CRP Monitoring Devices. Sci Rep.

[25] Piepenburg, O., Williams, C. H., Stemple, D. L. & Armes, N. A. DNA detection using recombination proteins. *Plos Bio*l 4, 1115–1121, doi:ARTN e20410.1371/journal.pbio.0040204 (2006). 10.1371/journal.pbio.0040204 PMC1475771 16756388

[26] Rodriguez NM, Wong WS, Liu LN, Dewar R, Klapperich CM (2016). A fully integrated paperfluidic molecular diagnostic chip for the extraction, amplification, and detection of nucleic acids from clinical samples. Lab on a Chip.

[27] Berney C, Danuser G (2003). FRET or no FRET: A quantitative comparison. Biophysical Journal.

[28] Millar JK (2005). DISC1 and PDE4B are interacting genetic factors in schizophrenia that regulate cAMP signaling. Science.

[29] Jin SL, Ding SL, Lin SC (2012). Phosphodiesterase 4 and its inhibitors in inflammatory diseases. Chang Gung Med J.

[30] Gobejishvili L (2013). Rolipram Attenuates Bile Duct Ligation-Induced Liver Injury in Rats: A Potential Pathogenic Role of PDE4. Journal of Pharmacology and Experimental Therapeutics.

[31] Suhasini AN (2016). A phosphodiesterase 4B-dependent interplay between tumor cells and the microenvironment regulates angiogenesis in B-cell lymphoma. Leukemia.

[32] Lee J, Samson AA, Song JM (2017). Inkjet-Printing Enzyme Inhibitory Assay Based on Determination of Ejection Volume. Anal Chem.

[33] Bender AT, Beavo JA (2006). Cyclic nucleotide phosphodiesterases: Molecular regulation to clinical use. Pharmacological Reviews.

[34] Kwak HJ (2005). Roflumilast inhibits lipopolysaccharide-induced inflammatory mediators via suppression of nuclear factor-kappaB, p38 mitogen-activated protein kinase, and c-Jun NH2-terminal kinase activation. J Pharmacol Exp Ther.

[35] Komatsu, K. *et al*. Inhibition of PDE4B suppresses inflammation by increasing expression of the deubiquitinase CYLD. *Nature Communications* **4**, doi:ARTN 168410.1038/ncomms2674 (2013). 10.1038/ncomms2674 PMC3644066 23575688

[36] Maurice DH (2014). Advances in targeting cyclic nucleotide phosphodiesterases. Nat Rev Drug Discov.

[37] Shakur Y (2002). Comparison of the effects of cilostazol and milrinone on cAMP-PDE activity, intracellular cAMP and calcium in the heart. Cardiovasc Drugs Ther.

[38] Schellekens RC, Stellaard F, Woerdenbag HJ, Frijlink HW, Kosterink JG (2011). Applications of stable isotopes in clinical pharmacology. Br J Clin Pharmacol.

[39] Schafer PH (2014). Apremilast is a selective PDE4 inhibitor with regulatory effects on innate immunity. Cellular Signalling.

[40] Zhang, C., Xu, Y., Zhang, H. T., Gurney, M. E. & O’Donnell, J. M. Comparison of the Pharmacological Profiles of Selective PDE4B and PDE4D Inhibitors in the Central Nervous System. *Scientific Report*s **7**, doi:ARTN 4011510.1038/srep40115 (2017). 10.1038/srep40115 PMC5215650 28054669

[41] MacKenzie SJ, Houslay MD (2000). Action of rolipram on specific PDE4 cAMP phosphodiesterase isoforms and on the phosphorylation of cAMP-response-element-binding protein (CREB) and p38 mitogen-activated protein (MAP) kinase in U937 monocytic cells. Biochemical Journal.

[42] Hatzelmann A, Schudt C (2001). Anti-inflammatory and immunomodulatory potential of the novel PDE4 inhibitor roflumilast *in vitro*. Journal of Pharmacology and Experimental Therapeutics.

