# Supplementary material for: Paper-based inkjet bioprinting to detect fluorescence resonance energy transfer for the assessment of anti-inflammatory activity
Source: Sci Rep. 2018 Jan 12;8:591. doi: 10.1038/s41598-017-18995-3 (PMC5766618; doi:10.1038/s41598-017-18995-3)
Supplement: Supplementary file 1 — Supplementary Tables [file 41598_2017_18995_MOESM1_ESM.pdf]

Paper-based inkjet bioprinting to detect fluorescence resonance energy transfer for the assessment of anti-inflammatory activity

Annie Agnes Suganya Samson, Jungmi Lee and Joon Myong Song*

College of Pharmacy, Seoul National University, Seoul 08826, South Korea

Supplementary Tables

Supplementary Table S1. Illustration of the solution densities of different reaction components.

S.No	Solution	ds in g/mL
1	Buffer	1.5296
2	cAMP	1.6073
3	PDE4B	1.5811
4	Eu- anti cAMP	1.5122
5	Ulight cAMP	1.5174
6	Roliparm	1.5602
7	Roflumilast	1.6003

Supplementary Table S2. Representation of ejection volume and their respective mole printed on the surface.

S.No	Reaction sample	Volume printed (nL)	Mole printed
1	cAMP (10 nM)	27.8	2.79E-16
2	PDE4B - High Conc. (10 µM)	28.06	2.81E-13
3	PDE4B - Medium Conc. (5 µM)	28.06	1.40E-13
4	PDE4B - Low Conc. (0.5 µM)	28.06	1.40E-14

Supplementary Table S3. Illustration of the number of mole of PDE4B inhibitors printed per spot area and their respective IM_{50} values.

C value	0	2 - 8	12 - 18	22 - 28	32 - 38	42 - 48	52 - 58	62 - 68	72 - 78	82 - 88	IM_{50}
**Roliparm**	0	0.29	0.32	0.62	1	1.33	1.75	2.11	2.43	2.72	2.46×10^{-13}mole
**Roflumilast**	0	0.04	0.07	0.38	0.77	1.1	1.5	1.9	2.2	2.5	1.86×10^{-13}mole
